# Local and long-range phase-amplitude coupling in a cortical spiking network model

**DOI:** 10.1186/1471-2202-15-S1-P222

**Published:** 2014-07-21

**Authors:** Peter W Donhauser, Sylvain Baillet

**Affiliations:** 1 McConnell Brain Imaging Center, Montreal Neurological Institute, McGill University, Montreal, Canada

## 

Phase-amplitude coupling (**PAC**) between neural oscillations in different frequency bands has been observed in population recordings of the hippocampus and neocortex [1,2]. Specifically, **bursts of gamma** (>30 Hz) oscillations are often found to be phase-locked to a slower oscillation in the **theta** range (4-8 Hz), raising questions about the mechanisms and computational consequences of this pattern. Recently, inter-areal gamma synchronization was also found to be systematically modulated by theta rhythms [3]. We study mechanisms for these experimental findings and their potential role in long-range inter-areal coupling using spiking neuron network models.

Using dynamical systems theory, bursting has previously been analyzed in detail for single neuron dynamics [4], requiring interaction of fast and slow adapting variables, driving the neuron through alternations of active (limit cycle) and resting (stable equilibrium) periods. On the population level, bursting can be studied within the same framework, treating average synaptic activity and time courses as slow/fast variables. Our model (Panel A) is based on observations of dynamically distinct interneuron populations in the cortex, namely fast-spiking, non-adapting (**FS**) and adapting interneurons (low-threshold spiking, **LTS**) exhibiting fast and slow synaptic time courses [5]. Coupled with a population of principal cells (regular spiking, **RS**), this network displays spontaneous phase-amplitude coupling as sparsely synchronized population bursts. Feed-forward connections between populations of this type lead to theta modulated gamma synchronization (Panel B and C).

We study in detail the parameter range/bifurcations of this model. Considering that in-vivo neural circuits are generally only sparsely synchronized, we contrast oscillatory patterns on the population level with stochastic firing of single neurons.

**Figure 1:**
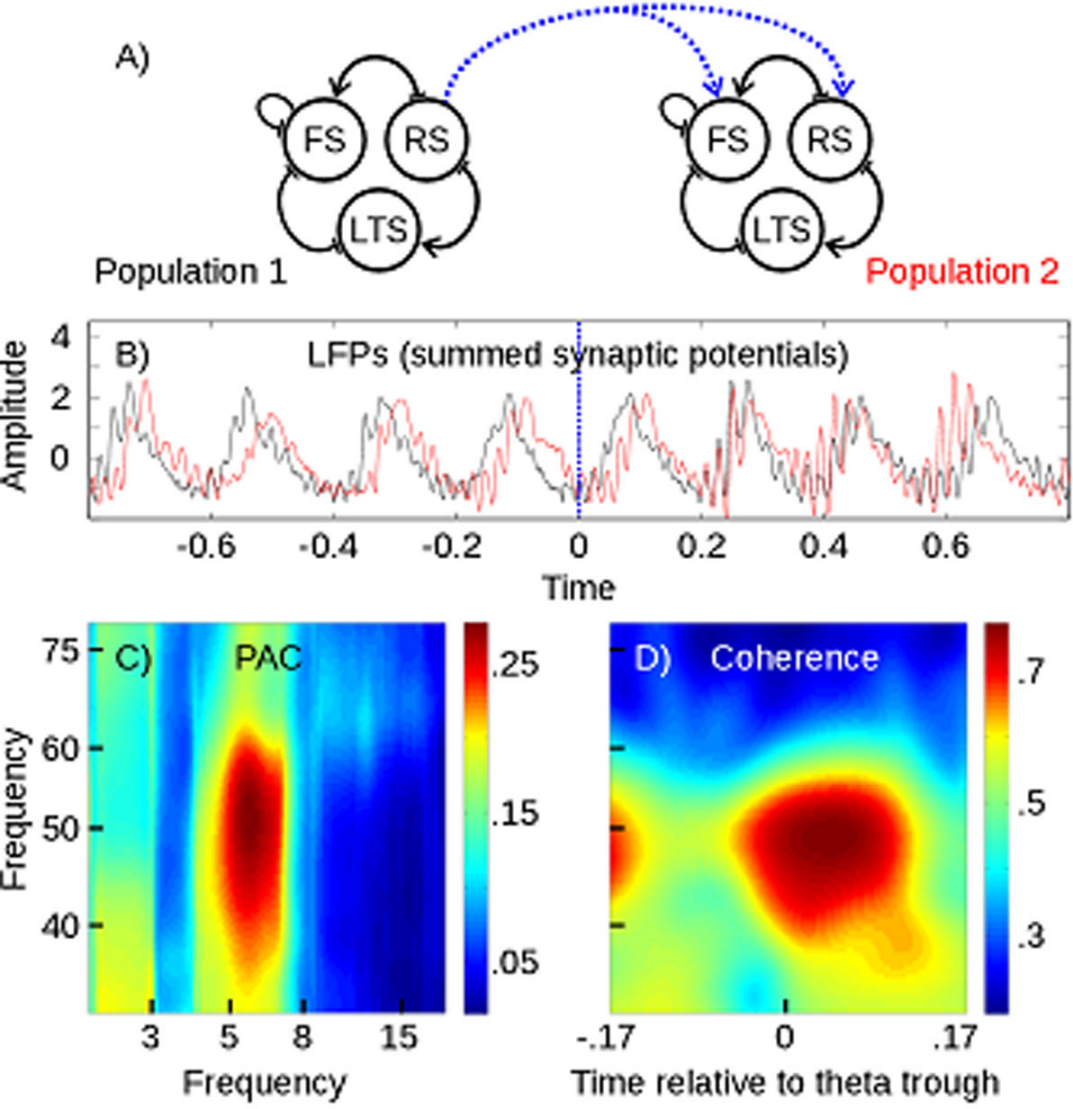
**Local and long-range phase-amplitude coupling in a cortical spiking network model** A) Schematic showing two populations of 1,000 spiking neurons and connections on the population level. Intra-/inter-areal connections are shown in black/blue respectively. B) Example of simulated LFPs, long-range connections are set at time-point zero. C) Spontaneous PAC in Population 1 (metric: mean-vector length as in [1]). D) Theta-modulated gamma coherence between Population 1 and 2.
